# Predicting Response to Neoadjuvant Chemotherapy with PET Imaging Using Convolutional Neural Networks

**DOI:** 10.1371/journal.pone.0137036

**Published:** 2015-09-10

**Authors:** Petros-Pavlos Ypsilantis, Musib Siddique, Hyon-Mok Sohn, Andrew Davies, Gary Cook, Vicky Goh, Giovanni Montana

**Affiliations:** 1 Department of Biomedical Engineering, King’s College London, London SE1 7EH, United Kingdom; 2 Department of Cancer Imaging, King’s College London, London SE1 7EH, United Kingdom; Rajiv Gandhi Centre for Biotechnology, INDIA

## Abstract

Imaging of cancer with ^18^F-fluorodeoxyglucose positron emission tomography (^18^F-FDG PET) has become a standard component of diagnosis and staging in oncology, and is becoming more important as a quantitative monitor of individual response to therapy. In this article we investigate the challenging problem of predicting a patient’s response to neoadjuvant chemotherapy from a single ^18^F-FDG PET scan taken prior to treatment. We take a “radiomics” approach whereby a large amount of quantitative features is automatically extracted from pretherapy PET images in order to build a comprehensive quantification of the tumor phenotype. While the dominant methodology relies on hand-crafted texture features, we explore the potential of automatically learning low- to high-level features directly from PET scans. We report on a study that compares the performance of two competing radiomics strategies: an approach based on state-of-the-art statistical classifiers using over 100 quantitative imaging descriptors, including texture features as well as standardized uptake values, and a convolutional neural network, 3S-CNN, trained directly from PET scans by taking sets of adjacent intra-tumor slices. Our experimental results, based on a sample of 107 patients with esophageal cancer, provide initial evidence that convolutional neural networks have the potential to extract PET imaging representations that are highly predictive of response to therapy. On this dataset, 3S-CNN achieves an average 80.7% sensitivity and 81.6% specificity in predicting non-responders, and outperforms other competing predictive models.

## Introduction

Neoadjuvant chemotherapy (NC) for cancer treatment is often given as a first step before the definitive surgery of a tumor, in order to facilitate surgical resection and improve the likelihood of a R0 resection [1], i.e. where there is a clear surgical margin on the pathological specimen. NC has been associated with improved survival after surgery for patients who respond to the therapy, and is considered the standard of care in some cancers [2, 3]. On the other hand, for patients who do not respond to NC, the prognosis after therapy is generally worse compared to primarily surgical approach alone [4]. When NC is not effective, it has also the disadvantage of exposing patients to unnecessary toxicity and may lead to adverse events while substantially increasing the associated health care costs and delaying definitive treatment. Identifying novel, non-invasive approaches for pretherapy prediction of NC response therefore holds the promise to stratify patients for preoperative therapy and has the potential to substantially improve the clinical outcome for certain patient populations or at least individualize treatment regimes.

Positron emission tomography (PET) is a nuclear medicine imaging technique based on the measurement of gamma rays resulting from positron emission using radiolabelled tracer molecules. These radiotracers allow biological processes to be measured and whole body images to be obtained which demonstrate sites of radiotracer accumulation. One of the most common radiotracers in use today is ^18^F-fluorodeoxyglucose (^18^F-FDG), a radiolabelled sugar (glucose analog) molecule. Imaging with ^18^F-FDG PET is used to determine sites of abnormal glucose metabolism and can be used to localize and characterise many types of tumor non-invasively. There is extensive evidence in the literature indicating the importance of ^18^F-FDG PET imaging in accurately characterizing disease, as well as determining stage and sites of recurrent disease in many cancer types [5]. For these indications, functional imaging with PET provides unique information which is not generally available from other standard medical imaging modalities such as CT and MRI.

Despite early indications that ^18^F-FDG PET imaging may also be a viable approach for predicting NC response using pre-treatment imaging [6], only a handful of quantitative measurements or biomarkers carrying predictive power have been found to be clinically useful. Some evidence has been reported that the amount of FDG uptake on pretreatment scans, as measured by tumor metabolic concentrations known as Standardized Uptake Values (SUVs) may carry predictive power [7–9]. The rationale for this approach is that the elevated FDG uptake in malignant cells is hypothesized to be associated with biologically relevant features, such as perfusion, cell proliferation, tumor viability, aggressiveness, and hypoxia [10–12], which are predictive of resistance to chemotherapy. However, SUV measurements are significantly affected by the initial ^18^F-FDG uptake kinetics and radiotracer distribution, which depend on the initial radiotracer injected activity as well as on the time elapsed between the tracer injection and the image acquisition. These factors can complicate the interpretation of SUV measurements due to their significant intra- and inter-observer variability [13]. For these reasons, and the fact that response prediction is not sufficiently accurate to use in the clinic, SUV measurements so far have been proved to be most useful in studies investigating the role of PET imaging to track ^18^F-FDG uptake changes over the course of an existing treatment [14, 15] rather than in predicting response from a single PET scan prior to therapy.

A particularly promising research direction that could potentially overcome the above limitations consists of the high-throughput extraction of large amounts of imaging features that can be made in direct relationship with clinical endpoints of interest. Radiomics [16, 17], an emerging field of research concerned with this objective, can potentially have a large clinical impact, since imaging is routinely used in clinical practice world-wide. In PET imaging, there has been increasing interest in identifying imaging features that characterize the spatial distribution and heterogeneity of ^18^F-FDG uptake patterns within a tumour by image analysis [18, 19]. This heterogeneity is hypothesized to originate in a number of physiological factors such as tumor metabolism, necrosis, cellularity, and angiogenesis, amongst others, and variability in these factors has been associated with more aggressive cancer behaviour, poorer response to treatment and worse prognosis [10–12]. The dominant methodologies for obtaining quantitative descriptors of spatial heterogeneity rely on texture analysis [20]. Such techniques encompass a broad range of mathematical descriptors that can be used to evaluate the spatial variation of voxel intensities both within a single PET slice as well as between adjacent slices, thus providing measures of intra-lesional heterogeneity. In contrast to SUVs, these descriptors provide a more accurate spatial characterization of FDG uptake patterns, and can potentially capture more signal. Whilst recent studies have started to explore the benefits of texture analysis for predicting response to NC therapy [21–25], drawing definite conclusions is difficult as each study relies upon different definitions of texture and deploys different predictive models. Another limiting factor characterising existing studies has been the small sample sizes, typically ranging from 10 to 50 patients.

The objective of this work is two-fold. First, we set out to explore whether a machine learning algorithm would be able to infer a predictive representation of a cancer’s metabolic profile, as captured by ^18^F-FDG PET imaging, in a larger patient population. In very recent years, biologically-inspired convolutional neural networks (CNN) have shown the ability to learn hierarchically-organised, low- to high-level features from raw images [26, 27], and yield state-of-the-art performance in the classification of both natural and medical images [28–31]. To investigate this question, we propose a neural network to harness the predictive power of spatially-varying ^18^F-FDG PET uptake patterns. The proposed architecture, 3S-CNN (three-slices convolutional neural network), produces features that are representative of metabolic activity in cancer, before therapy, and we expect that this method would ultimately distinguish responders to non-responders. Our second objective is to compare the performance of 3S-CNN with competing predictive algorithms where the quantitative tumour phenotypes are represented by over 100 “hand designed” texture features, capturing patterns in both two- and three-dimensions, as well as SUV summaries. Whereas previous studies have each reported on the performance of a very specific approach, generally combining a handful of selected texture features with a single statistical classifier, here we aim at a more comprehensive empirical characterization of a large battery of quantitative descriptors and predictive models. In this respect, our results set a comparative benchmark for future radiomics developments in this area.

## Material and methods

### Oesophageal cancer data

For this study we obtained a dataset consisting of *n* = 107 patients (83 males, 24 females) with newly diagnosed esophageal cancer at a tertiary referral centre, Guys and St Thomas NHS Trust (GSTT). The study was approved by the Westminster ethics committee, and all patient information was anonymized prior to analysis. The age at time of diagnosis ranged from 32 to 80 with an average of 62±25 years. All patients underwent pre-treatment whole-body ^18^F-FDG PET/CT for staging. For each patient, the primary tumor was positively identified on axial ^18^F-FDG PET images by an experienced nuclear medicine physician.

**Fig 1:**
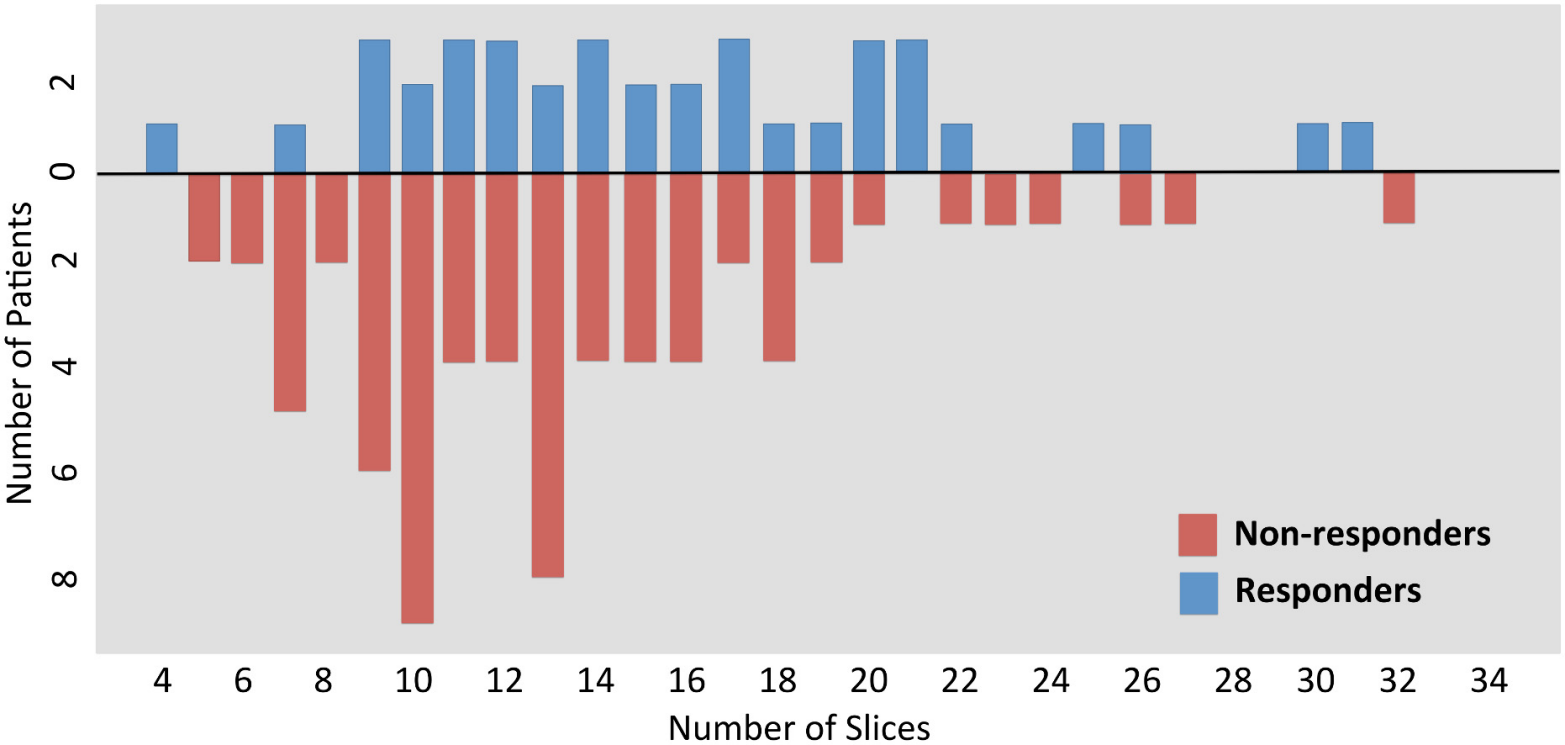
Distributions of axial ^18^F-FDG PET intra slices extracted from the 3D tumor volume of non-responders and responders.

**Fig 2:**
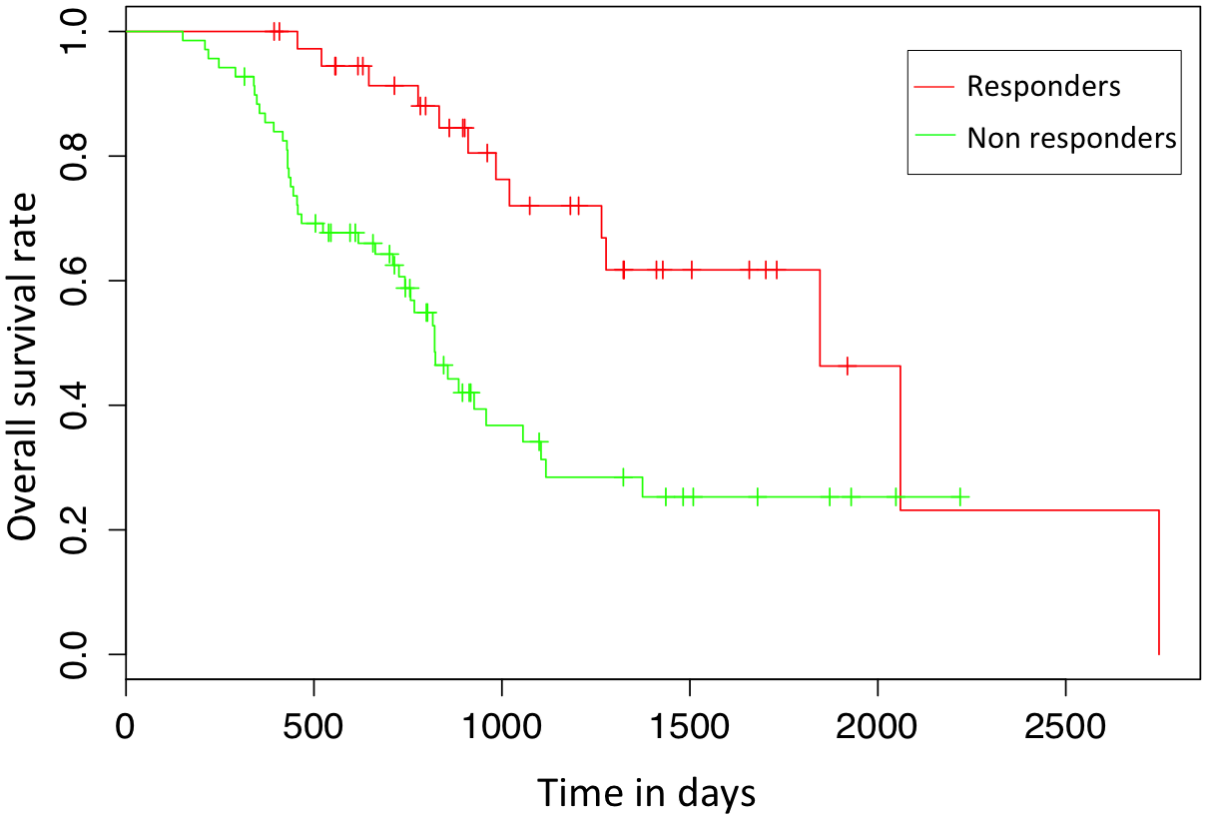
Kaplan-Meier plot showing the survival rates of responders and non-responders.

Bespoke software was developed for the automatic delineation of the tumor ROIs. This was achieved by applying a 40% slice-wise maximum intensity threshold to exclude voxels with less than 40% of the activity in the voxel of maximum intensity within the same axial slice. Using this approach we were able to accurately delineate the regions of high ^18^F-FDG uptake across all tumors in the study. Each pixel corresponded to a voxel size of 4.7 × 4.7 *mm* with 3.27 *mm* slice thickness, and the size of the slices in the dataset varies from 13 × 16 to 93 × 79 pixels. Also, as can be observed in Fig 1, there is a wide inter individual variability in the number of axial slices which were extracted from the 3D tumor volumes. The number of axial slices per tumor varied from 4 to 32. 86 tumors were adenocarcinoma, 20 were squamous cell carcinoma, and one was undefined. Nearly half of the patients had a moderately differentiated tumour (58). 70 patients had a T3 primary lesion, 80 had N1 lymph node metastasis, and 1 patient had distant metastasis. All patients were treated with neoadjuvant chemotherapy, and 38 responded to treatment. The response was assessed using a pathological Mandard tumor regression grade [32]. For the purpose of this study, we grouped the patients into two distinct pathologic groups: the non-responders group, which includes subjects whose tumor showed no regressive changes (Grades 4 and 5), and the responders group, which includes all those cases in which regressive changes were noted (Grades 1, 2 and 3) as originally proposed in [32]. Among the responders, 26 (68.4%) had TNM clinical stage III; among the non-responders, 20 (29.0%) had clinical TNM stage II. The overall survival (OS) period was defined as the time in days between the PET scan and the date of death. Responders to therapy had a median OS of 972.5 days compared with 714 days for non-responders. Fig 2 illustrates the overall survival rates, which were found to be significantly different by a Kaplan-Meier analysis (p-value = 0.00045).

### Texture analysis

Texture analysis refers to a variety of mathematical methods used to provide information about the spatial arrangement of voxel intensities within a volume neighborhood containing the tumor [21, 25]. In our study, we used texture analysis to characterize the 3D uptake heterogeneity in a tumor that is captured by the PET scans. We employed two broad classes of texture feature extraction techniques, statistical- and model-based. The statistical approach consisted of quantifying some aspects of the spatial distribution of voxel values by taking into account local features at each point in the image, and extracting a set of statistics from the distributions of these features.

The statistics-based approach relies on first-, second- and higher-order statistics. First-order statistics were calculated from the original voxel intensity values without taking into consideration the relationship of each voxel with its neighbors. This class encompassed measures of central tendency (including mean, median, mode, percentiles, quartiles), variability (including range, interquartile range, variance, standard deviation, coefficient of variation, skewness, and kurtosis), first order energy, and entropy. Second order statistics consist of co-occurence measurements between two pixels calculated using both Gray-Level Co-occurrence Matrices (GLCMs) [33] and Gray-Level Difference Matrices (GLDMs) [34]. GLCMs determine how many times a voxel with a given intensity occurs jointly with another voxel having a different intensity, whereas GLDMs are based on absolute differences between pairs of voxel intensities. Higher order statistics capture properties of three or more voxel values occurring at specific locations relative to each other, and are extracted from Gray-Level Run Length Matrices (GLRLMs) [35], Gray-Level Size Zone matrices (GLSZMs) [36] and Neighborhood Gray-Tone Difference Matrices (NGTDMs) [37]. Both GLRLMs and GLSZMs analyze texture in a specific gray-level run and zone, respectively. Gray-level run is the length of consecutive voxels having the same intensity in a preset direction in the image, whereas the zone is a cluster of consecutive voxels having the same intensity. A GLRL matrix is a two-dimensional matrix in which each element *p*(*i*, *j*∣*α*) gives the total number of occurrences of runs of length *j* at gray level *i* in a specific direction *α*. Following the same principle, the entries of a GLSZ matrix are the number of clusters of size *s* of gray-level *i*, where the size of a cluster is defined as the number of consecutive pixels with gray-level *i*. Finally, NGTDM are column matrices that describe the differences between each voxel and its neighboring voxels in adjacent image planes, and are thought to closely resemble the human experience of the image [37]. In NGTDM, the *i* ^*th*^ entry is a summation of the differences between all pixels with gray-tone *i* and the average value of their surrounding neighbors.

Model-based approaches used mathematical models such as fractal analysis to represent texture information. Fractal analysis is a form of geometric pattern recognition that evaluates the self-similarity and roughness of a surface at different levels [38, 39]. Such evaluation in the context of PET imaging can quantify the ^18^F-FDG uptake heterogeneity of a tumor volume [40, 41]. A fractal is defined as a set for which Hausdorff-Besicovich dimension is strictly greater than the topological dimension [42]. The fractal dimension (FD) is the defining property in the study of texture analysis. The fractal dimension of each voxel of the ^18^F-FDG uptake is calculated inside a moving window centred on the voxels by using a differential box-counting method [43]. A summary of the texture matrices and the extracted texture features we have calculated is provided in Table 1. In total we considered 85 texture features, on top of which we then added 18 statistical summaries of SUV measurements (including minimum, maximum, mean). By including all the above features, for each tumor volume *i*, we end up extracting a quantitative descriptor of the ROI consisting of a 103-dimensonal vector denoted $t_{i}=\left(t_{i,1},\cdots ,t_{i,103}\right)$, for *i* = 1, 2, …, *n*.

**Table 1:** Summary of second and high order texture features extracted from texture analysis.

Texture Matrices	Texture Features
**Gray Level Co-occurence**	Energy, Autocorrelation,
	Cluster Prominence, Cluster shade,
	Contrast, Correlation, Difference Entropy,
	Difference Variance, Dissimilarity,
	Entropy, Homogeneity, Difference Moment
	Information Measure Cor.1/Cor.2,
	Sum Average, Sum Entropy, Sum Variance
	Inverse Difference Moment Normalized,
	Inv. Difference. Normalized, Max. Probability,
**Gray Level Run Length**	Short Run Emphasis, Long Run Emphasis,
	Short Run Low/High Gray Level Intensity,
	Long Run High/Low Gray Level Intensity,
	Run Length Non-uniformity,Run Percentage,
	Intensity Variability, Run Length Variability
	High/Low Gray Level Run Emphasis,
**Gray Level Size Zone**	Short/Long Zone Emphasis, Zone Percentage,
	Short Zone Low/High Emphasis,
	Long Zone High/Low Emphasis,
	Intensity Non-uniformity,
	Zone Length Non-uniformity,
	Low/High Intensity Zone Emphasis,
	Intensity Variability, Size zone Variability
**Gray Level Difference**	Mean, Entropy, Variance, Contrast
**Fractal Based Features**	Mean, Standard Deviation,
	Hurst Exponent, Lacunarity
**Neigh. Gray Tone Difference**	Coarseness, Contrast, Busyness,
	Texture Strength, Complexity

The individual feature vectors **t**_i_ obtained for the pretherapy PET scans were used as predictors to train and test state-of-the art machine learning techniques for the prediction of NC therapy response. The objective was to minimize the classification error on unseen test data. We considered four different statistical classifiers: logistic regression (LR) [44], gradient boosting (GB) [45], random forests (RF) [46], and support vector machines (SVM) [47]. Logistic regression is a common linear method for multi-variable modeling of binary outcomes. Both GB and RF embrace the notion of ensemble learning, whereby an entire collection of learning algorithms is deployed in order to obtain superior predictive performance. More explicitly, GB algorithm builds an ensemble of regression trees in a stage-wise fashion, where each one is trained with respect to the error of the whole ensemble learnt so far. On the other hand, the RF algorithm builds an ensemble of de-correlated classification trees, where each one is trained on a random subsample of the training dataset and then combines the trained classification trees by averaging their probabilistic prediction. Finally, the kernel-based SVM algorithm discriminates between responders and non-responders using hyperplanes that maximize the margin between the two classes in a non-lineal feature space. The key idea of kernels is to project the input explanatory variables of our dataset into high dimension hyperplanes where the discrimination between responders and non responders is improved.

### Convolutional neural networks

A Convolutional Neural Network (CNN) is a special feed-forward neural network for learning a hierarchical representation of imaging data [26, 27] and then using these representations for imaging recognition tasks. In traditional classifiers like LR, SVM, GB and RF, there is a need for preprocessing the images and to extract texture features relevant to a specific task. The limitation of these classifiers originates from the fact that the performance is highly dependent on the design of the texture features, thus requiring prior knowledge for a specific task and expertise in hand-engineering the necessary features. By contrast, CNN operates directly on raw images and attempts to automatically extracts highly expressive imaging features relevant to a specific task at hand.

Compared to standard neural networks, the individual neurons in a CNN are tiled in such a way that they respond to overlapping portions of the input image. The main architectural components of CNN are the convolutional and subsampling layers. The neurons of the convolutional layer receive information from only a subset of the inputs, called receptive field. As a result, each neuron learns to detect features from a local region of the input image. This allows us to capture the local substructure and preserve the topology of the input image. In addition to local connectivity, a convolutional layer also imposes groups of neurons, whose receptive fields are located in different places of the input image, to share exactly the same weight values. The outputs of these groups of neurons are called feature maps. The technique of the weight sharing reduces the number of free parameters, thus increasing the generalization ability of the network [48]. A convolutional layer is composed of several feature maps, so that a rich variety of imaging features can be extracted. The convolutional layers are then followed by subsampling layers whose purpose is to reduce the dimensionality of the convolutional responses by selecting superior invariant imaging features. In an attempt to achieve a distributed and more abstract representation of the input image, multiple convolutional-subsampling layers are stacked on top of one another, thus delivering a deep architecture of multiple non-linear transformations. Each layer generates a representation of the image based on the feature-detecting role of the neurons. By stacking layers of feature-detecting neurons, a CNN is able to infer highly expressive representations carrying predictive power for imaging recognition tasks [28].

**Fig 3:**
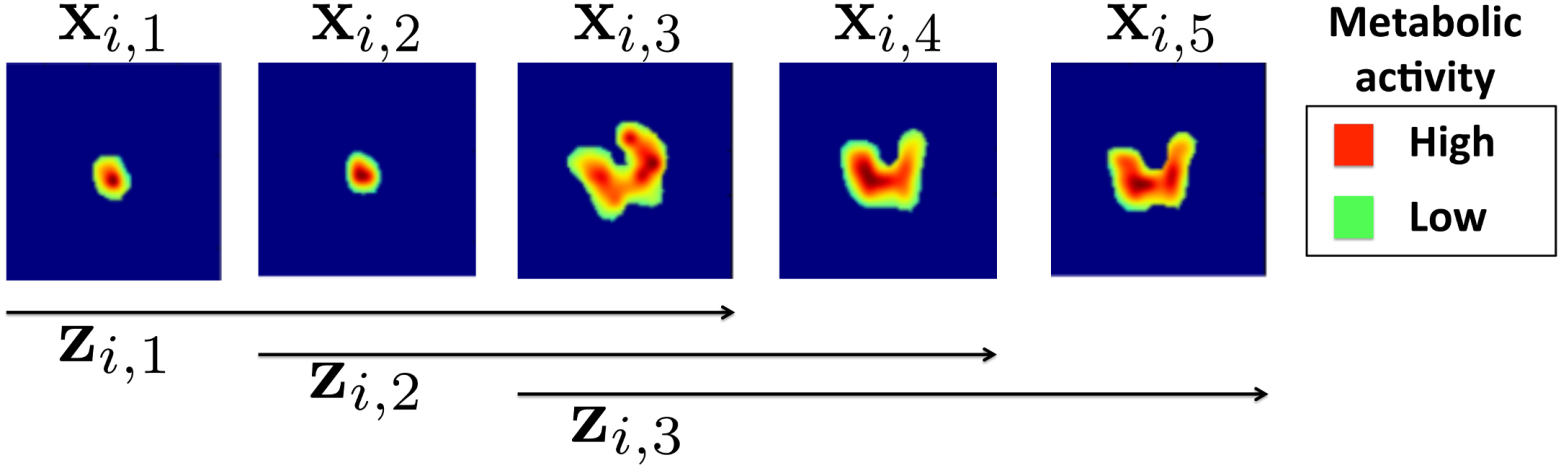
^18^F-FDG PET ROIs of a specific tumor *i* after segmentation embedded into larger square background of standard size of 100 × 100 pixels. Each enlarged slice is denoted by **x**_*i,j*_ and each set of three spatially adjacent enlarged slides is denoted by **z**_*i,k*_, where *j* and *k* represent the slices and triplets of the specific tumor *i*. In this example only 3 triplets, from the 5 available slices can be formed, so *k* = 1,2,3.

In our application the object to be classified is a ROI representing the tumor, which has a three-dimensional shape. Using ROIs as direct input of the CNN is infeasible due to the fact that every tumor has a different shape and size. To address this issue, we initially embedded all individual ROIs into 3D cuboids of standard width and length, and height varying according to the number of slices of each ROI. Specifically, each 2D intra-tumor slice was embedded into a larger and squared background of standard size 100 × 100 pixels, which was sufficiently large to include all the observed tumors (see Fig 3). For a given tumor *i* having *m*_*i*_ slices, we denote each enlarged slice as **x**_*i,j*_ where *i* = 1, 2, …, *n* and *j* = 1, 2, …, *m*_*i*_. We denote the entire standardized volume containing the tumor for patient *i* as **x**_*i*_. Our assumption is that a neural network architecture able to capture patterns of FDG uptake that occur within each 2D slice as well as across multiple adjacent slices may detect salient imaging features that are important for predicting chemotherapy response. Under this assumption, we propose an architecture that initially fuses the spatial information across adjacent intra slices. For a given standardized volume **x**_*i*_ containing *m*_*i*_ slices, we build all possible sets of three adjacent slices, which we denote as $z_{i,k}=\left\{x_{i,k},x_{i,k+1},x_{i,k+2}\right\}$ where *k* = 1, …, *m*_*i*_ − 2. This process is illustrated in Fig 3. Each triplet **z**_*i,k*_ was then treated as a three-channel input for the CNN. Associated with each triplet **z**_*i,k*_ there is a corresponding binary label, *y*_*i*, *k*_, indicating whether the patient has responded (*y*_*i*, *k*_ = 1) or not responded (*y*_*i*, *k*_ = 0) to therapy.

The first convolutional layer of the CNN, denoted **U** ^(1)^, consists of *R* ^(1)^ feature maps. Each feature map is obtained by convolving all slices within a triplet **z**_*i,k*_ with a weight matrix $k_{p,j}^{\left(1\right)}$, to which we then add a bias term $b_{j}^{\left(1\right)}$. The output is then passed through an hyperbolic tangent function *f*(⋅), i.e.

$$u_{j}^{\left(1\right)}=f\left(\sum_{p=0}^{2}k_{p,j}^{\left(1\right)}*x_{i,k+p}+b_{j}^{\left(1\right)}\right)\;j=1,\ldots ,R^{\left(1\right)}.$$

Each element of a feature map $u_{j}^{\left(1\right)}$ in the first convolutional layer enclose information from a local 3D tumor uptake region. The *R* ^(1)^ weight matrices, one for each feature map, are learned in order to build a library of low-level features which are extracted by inspecting various locations of the input triplet. Within each PET slice, the same weight matrix is convolved with the entire slice. This results in the weight being shared by many overlapping squared sub-windows of the slice, and also in sparse connections between the input units and the hidden units in the first layer.

Once each feature has been learned, its exact location within the triplet becomes less important, as long as its approximate position relative to other features is preserved. The convolutional layer is then followed by a subsampling layer which reduces the dimensionality of each feature map. This is achieved by retaining only the maximum value within each non-overlapping sub-region of size (2 × 2) for each feature map. This max-pooling operation is carried out in order to down-sample each feature map by a factor of 2 along each direction and improve generalization performance by selecting invariant features [49]. The max-pooling layer has the same number of output and input feature maps and does not require any additional parameters.

**Fig 4:**
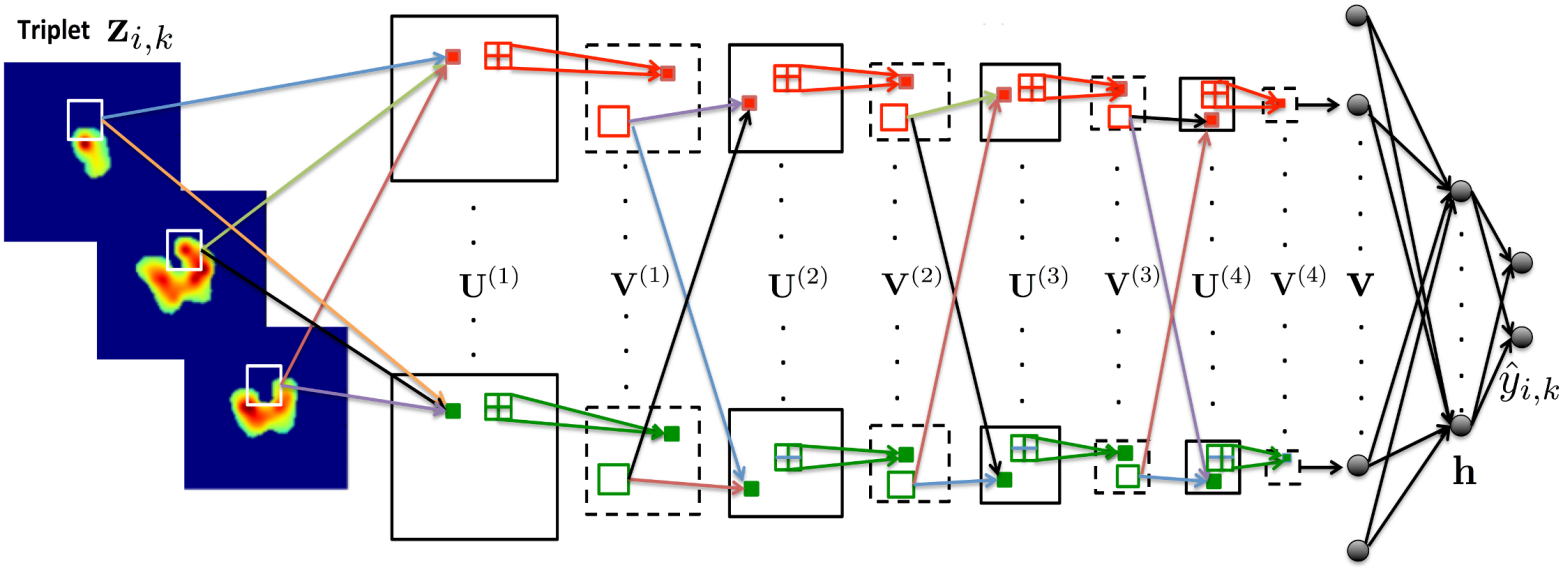
CNN architecture for fusion of 3 adjacent ^18^F-FDG PET intra slices into a vector v. The CNN architecture is composed from 4 convolutional and 4 max-pooling layers denoted by ${\left\{U^{\left(i\right)}\right\}}_{i=1}^{4}$ and ${\left\{V^{\left(i\right)}\right\}}_{i=1}^{4}$. In the first convolutional layer **U** ^(1)^, different coloured arrows represent the usage of different learnable weight matrices for convolving each PET slice in the triplet. Colored dotted rectangles in the feature maps represent elements of the feature maps that enclose local spatial information of the previous layer in the architecture. In the Max-pooling layers 2 × 2 element windows represent non-overlapped grids from which we choose the maximum element to downsample the feature maps.

In order to extract higher-level features from the low-level features obtained in the initial layers, additional convolutional layers are added, which are always followed by a pooling layer. Each additional convolutional layer spans all the pooled feature maps obtained at the previous layer (see Fig 4). For instance, each feature map in the second layer is obtained as

$$\begin{array}{c} u_{j}^{\left(2\right)}=f\left(\sum_{p=1}^{R^{\left(1\right)}}k_{p,j}^{\left(2\right)}*v_{p}^{\left(1\right)}+b_{j}^{\left(2\right)}\right)\;j=1,\ldots ,R^{\left(2\right)}. \end{array}$$

The complete architecture contains four convolutional/max-pooling layers (see Fig 4). The resulting set of max-pooled feature maps $v_{j}^{\left(4\right)}$, *j* = 1, 2, …, *R* ^(4)^ enclose the entire spatial local information as well as the rich hierarchical representation of the input triplet **z**_*i,k*_. Each feature map $v_{j}^{\left(4\right)}$ is then flattened out and all the elements are collected into a single vector **v** of dimension *R*. These units provide the input for a fully connected (FC) hidden layer, **h** consisting of *H* units. The activation of the *j* ^th^ unit of the FC layer is given by

$$\begin{array}{c} h_{j}=f\left(\sum_{k=1}^{R}M_{kj}v_{k}+b_{j}\right),\;j=1,\ldots ,H. \end{array}$$

All weights are collected into a matrix **M**. The probability that each **z**_*i,k*_ is assigned to class 1 (responder) is given by the soft-max function

$$\begin{array}{c} p\left({\hat{y}}_{i,k}=1|h;\theta_{1},\theta_{2}\right)=\frac{\exp\{\theta_{1}h\}}{\sum_{j=1}^{2}\exp\left\{\theta_{j}h\right\}}, \end{array}$$

where the vectors $\theta_{1}$ and $\theta_{2}$ are the columns of the softmax matrix $\Theta_{H\times 2}$ According to this rule, a triplet **z**_*i,k*_ is assigned to class 1 when $p({\hat{y}}_{i,k}=1)>0.5$. In case of ties, we take the prediction as being wrong.

In order to predict whether an unseen tumor volume **x**_*i*_ respond or not to the therapy, we use a majority vote rule based on the estimated prediction probabilities for all triplets extracted from the tumor. We predict that **x**_*i*_ is a responder when

$$\begin{array}{c} \frac{1}{m_{i}-2}\sum_{k=1}^{m_{i}-2}\text{I}\left({\hat{y}}_{i,k}=1\right)>0.5, \end{array}$$

where I(⋅) is an indicator function that is 1 when ${\hat{y}}_{i,k}=1$ and otherwise is zero.

The parameters of the CNN consists of all the convolutional weights $k_{j,R_{l}}^{\left(l\right)}$, the weight matrix **M** and the soft-max parameter matrix $\Theta_{H\times 2}$. These unknown parameters, denoted by **W**, are learned by minimizing the negative log-likelihood function,

$$\begin{array}{c} \ell \left(W\right)=-\sum_{i=1}^{n}\log\left(p\left({\hat{y}}_{i,k}=y_{i,k}|z_{i,k},W\right)\right). \end{array}$$ (1)

In our experiments we used a stochastic gradient-descent algorithm with mini batches (MSGD). MSGD is a variant of the gradient descent algorithm commonly used to train neural networks on large datasets [50]. At each update of the weights in the SGD algorithm, instead of considering all the training data to compute the gradient of the loss function ℓ, only one small batch of training data at a time is used. We also take advantage from the parallelization of the MSGD algorithm in order to accelerate the training of our CNN on GPU cards. Our code is based on Theano, a Python library that compiles symbolical expressions into C/CUDA code for deployment on both CPUs and GPUs [51].

### Comparative analysis

We carried out a comparative analysis of different machine learning algorithms for NC therapy response prediction. As well as the 3S-CNN model, which takes sets of intra-tumor triplets as input, we also implemented a simpler 1S-CNN architecture that treats each individual slice **x**_*i,k*_ as an independent sample, and eventually make a decision based on a majority vote rule, exactly as in 3S-CNN. This simpler architecture is added here to study the potential advantages deriving from exploiting inter-slice patterns that capture 3D information as in 3S-CNN. The performance of all comparable algorithms were obtained by averaging the outcome of three independent experiments. Each experiment was conducted using a different combination of training and test sets. In each case, 96 patients were assigned to the training set, and the remaining 11 were utilized for testing.

For the 3S-CNN architecture, each training set consisted of all triplets of adjacent ^18^F-FDG PET slices extracted from the tumors, and each triplet was treated as a training example. Furthermore, in order to create more training examples and prevent our CNN model from overfitting, the training set was artificially augmented by rotating each triplet by *κ*⋅60°, *κ* = 1,2,3,4,5. Overall, we created a balanced training dataset of 5316 FDG-PET triplets for both responders and non-responders to therapy. Training of the 1S-CNN architecture was done in a similar way, whereby each slice within a tumour contributed a training example. For fair comparisons between CNN models, the same augmentation strategy was always used. For the purpose of tuning and optimising all the hyperparameters, which include the number of layers, weight matrices size, and number of feature maps in each layer and learning rate, 30% of the training dataset was used as validation set.

The predictive models trained on texture and SUV features are denoted LR, GB, RF, and SVM when using the original feature vectors, and LR-PCA, GB-PCA, RF-PCA and SVM-PCA when using the ten largest principal components extracted from the feature vectors. PCA was used to reduce the dimensionality of the input by a factor of 10 whilst retaining as much information as possible. For each model we deployed a grid search using 10-fold cross validation to choose the set of hyperparameters. In 10-fold cross-validation, the original training sample is randomly partitioned into 10 equal size subsamples. Of the 10 subsamples, a single subsample is retained as the validation data for testing the model, and the remaining 9 subsamples are used as training data. The cross-validation process is then repeated 10 times, with each of the 10 subsamples used exactly once as the validation data. The 10 results from the folds can then be averaged to produce a single estimation. For the SVM classifier we tested linear, polynomial and Gaussian kernels, and here report only on the best SVM performance, which was obtained by the polynomial kernel.

The forward stage-wise fashion of the GB allows us to automatically assess the contribution of each variable in the construction of a robust classification rule [52]. In the GB algorithm at each node of each regression tree a specific variable is used to partition the sample of patients associated with that node into subregions. The particular variable chosen is the one that gives maximal estimated improvement in squared error risk over that for a constant fit over the entire sample of patients. In each regression tree, the squared relative importance of this variable is the sum of such squared improvements over all the internal nodes for which it was chosen as the splitting variable. This importance measure can be generalized to the forward stage-wise expansion of regression trees of the GB algorithm by simply averaged over the trees which were induced in the ensemble.

We also examined the performance of a classifier based only on SUVmax measurements. The SUVmax summaries were thresholded by performing a receiver operating characteristic (ROC) analysis. The optimal threshold was identified by means of a grid search using values within the range of the SUVmax measurements extracted from the training dataset. From the ROC curve we chose the threshold associated to the maximum sum of true positive and true negative rates. Then we classified each of the remaining 11 patients as responders if the corresponding SUVmax measurement was below the threshold.

Finally, we explored a potential association between response to treatment and TNM staging and grading, as these two parameters are commonly adopted in clinical practice. TNM stages were divided into two groups, stage II and stage III, and the strength of their association was not found to be statistically significant (p-value = 0.73) using a Pearson’s *χ* ^2^ test. Analogously, a potential association between the grading system and response to therapy was tested by first lumping together well and moderately differentiated tumours into one group, and using poorly differentiated tumors as second group. Again, there was no evidence of a statistically significant association (p-value = 0.41).

## Experimental results

The performance metrics relative to all the predictive models are summarized in Table 2. Specificity represents the proportion of actual respondents (positives) which are correctly identified as such, and the sensitivity represents the proportion of non-respondents (negatives) which are correctly identified as such. In terms of average accuracy, the 3S-CNN algorithm outperforms all other models, and its performance is followed by a GB algorithm trained on hand-crafted features. Excluding LR, all the classifiers trained on texture features perform better when the feature vector is replaced by principal components. Finally, we note that apart from the 3S-CNN algorithm, all other algorithms were outperformed by the SUVmax median threshold.

**Table 2:** Classification results: each figure is the average of three independent experiments using different training and test datasets.

Method	Sensitivity	Specificity	Accuracy
**3S-CNN**	80.7±11.5	81.6±9.2	73.4±5.3
**1S-CNN**	77.9±12.9	58.3±4.2	66.4±5.9
**GB**	70.5±6.0	63.8±6.1	66.7±5.2
**GB with PCA**	68.1±7.9	46.8±16.2	66.8±6.0
**RF**	61.0±8.6	36.4±18.4	57.3±7.8
**RF with PCA**	65.8±7.5	52.0±28.9	65.7±5.6
**SVM**	66.9±8.5	38.4±19.2	55.9±8.1
**SVM with PCA**	67.4±10.3	50.9±5.0	60.5±8.0
**Logistic Reg**.	60.4±6.2	38.3±7.3	51.4±3.0
**Logistic Reg. with PCA**	58.9±4.9	38.9±12.5	48.4±8.0
**SUV max with threshold**	33.0±33.0	35.2±10.2	41.0±4.5
**SUVmax median threshold**	81.5±1.5	53.0±13.0	67.7±4.2

**Fig 5:**
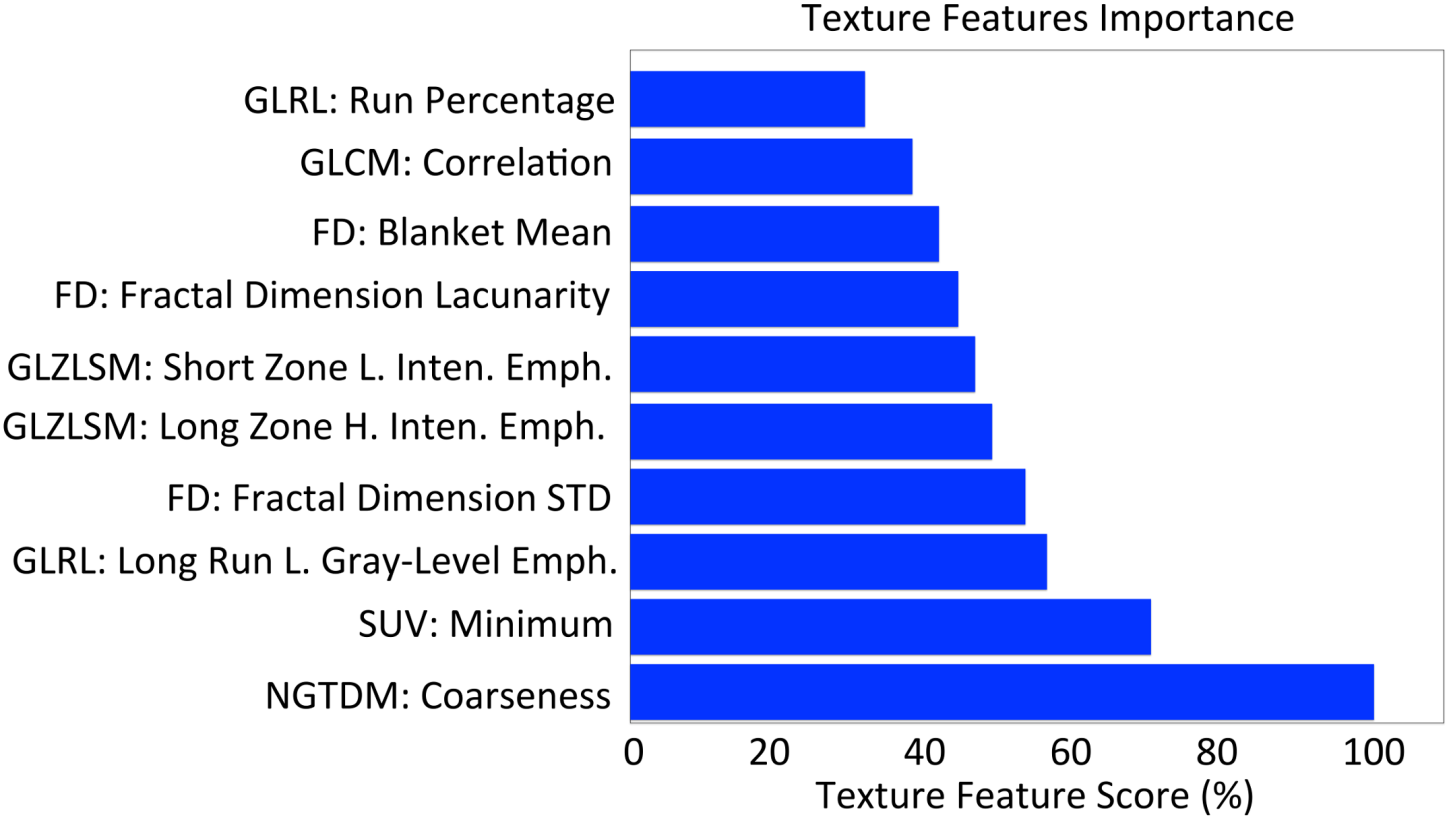
Ten most important texture features for prediction of the chemotherapy response using the GB algorithm. Since these measures are relative, we assign the largest importance a value of 100% and then scale the others accordingly.

Fig 5 reports the top 10 features and their corresponding score extracted from the GB algorithm. In this figure the feature with largest importance has been given a score of 100%, and all the other features have been scaled accordingly. From this feature ranking analysis it emerges that coarseness, which has been linked to granularity within an image, is the most important feature for response prediction. Coarseness describes local tumor texture based on differences between each voxel and the neighboring voxels in adjacent axial ^18^F-FDG PET images.

**Fig 6:**
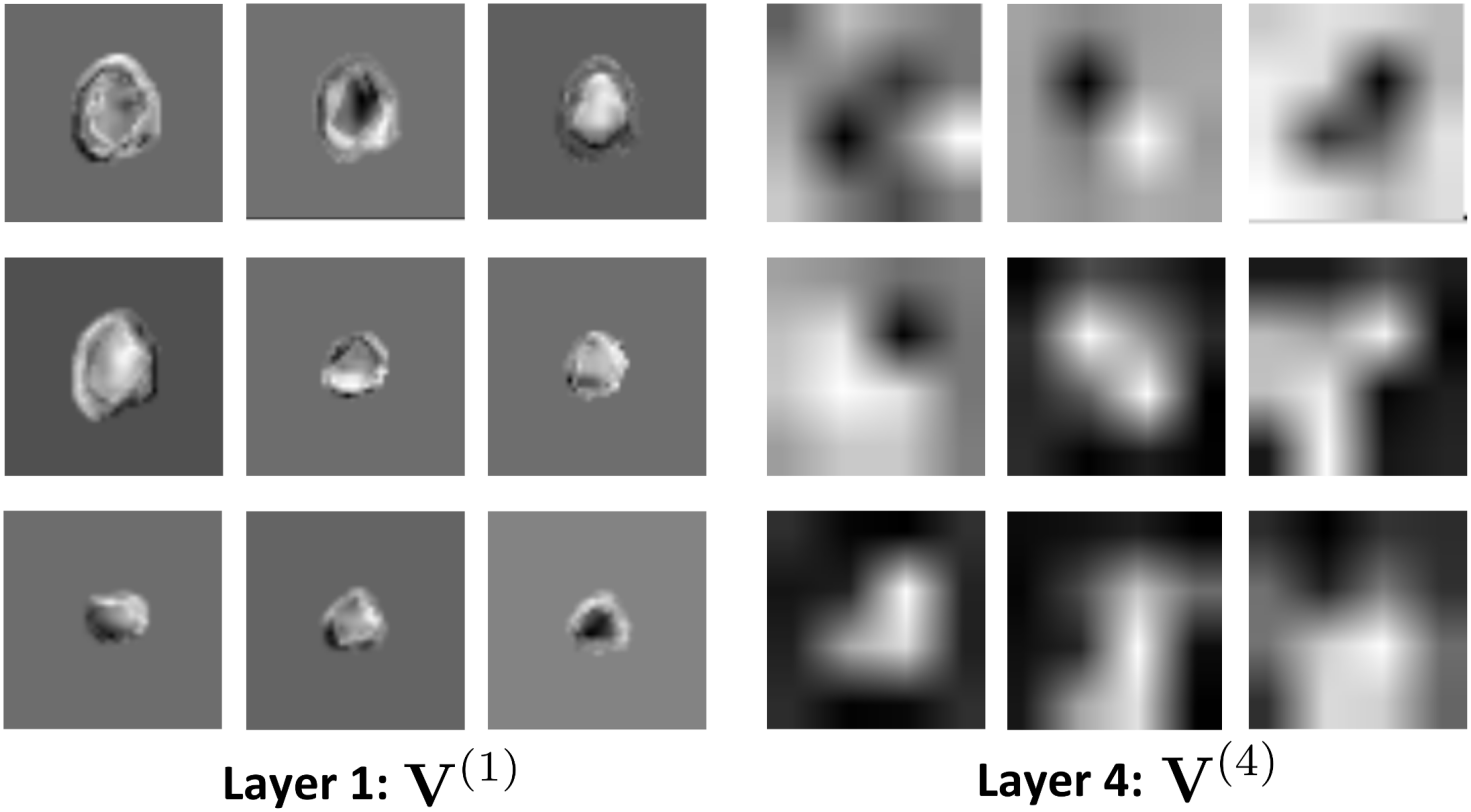
Examples of feature maps in the first and last max-pooling layers **V** ^(1)^ **,** **V** ^(4)^ of the CNN architecture. The feature maps illustrate how a specific triplet is represented in the first and last max-pooling layers.

In Fig 6, we illustrate feature maps from the first and last max-pooling layers **V** ^(1)^, **V** ^(4)^ of the CNN architecture. These feature maps demonstrate how a specific triplet is represented in the first and last max-pooling layers. The feature maps in the first layer appear to have fused the information from the three adjacent slices of the triplets. In the last layer, the 3S-CNN architecture represents the triplets by remarkably clear and well-defined geometrical patterns with the same level of metabolic activity.

## Discussion

The experimental results in Table 2 provide evidence that 3S-CNN outperforms the predictive algorithms trained on a large set of pre-determined imaging features. We believe that the features produced by this approach encompass several of the standard texture features, with the advantage of being completely automatic. The superior performance achieved by the CNN algorithm is due to the exploitation of 3D ^18^F-FDG uptake information captured by the PET scans and the fact that the architecture learns imaging features that are directly relevant to the clinical endpoint. To our knowledge, the potential predictive power of deep neural networks that only use the raw data as input, and build internal representations of the PET images, has never been assessed for the prediction of chemotherapy response.

In the literature, the decrease in mean or maximum metabolic activity measured by SUV parameters within the first two weeks of neoadjuvant therapy is often considered to be the best available predictor of histopathologic tumor response, however the sensitivities and specificities are still below 67 − 70% (95% confidence intervals ranging from 62% to 76%) [53, 54]. Beyond the prognostic role of FDG uptake changes over the course of the treatment, the role of SUV parameters from only baseline ^18^F-FDG PET images has been investigated in various studies [7–9], but results conflict as to whether SUV parameters carry any predictive power to assess response to therapy. Specifically, two studies reported that patients with high initial SUVmax values associated with higher probability of response to chemoradiation while one study reported that SUV measurements were not significant factors of the response.

Two studies have explored the capacity of textural features extracted from only pretherapy PET images to differentiate patients with respect to response to therapy. Tixier et al. [21] have analyzed the association between 38 textural features extracted from pretherapy ^18^F-FDG PET images of 41 patients with esophageal cancer and response to therapy using the Kruskal-Wallis non-parametric test. The sensitivity and specificity reported here varied from 46% to 92% and 45% to 91%, respectively. The predictive capacity of SUV parameters and textural features extracted from GLCMs and NGTDMs has been investigated by Cheng et al. [55] in a cohort of 70 patients with esophageal cancer. This study reported AUC (area under the curve) values of 0.662 for SUV entropy and 0.663 for uniformity. Finally, several studies have investigated the role of textural features and SUV parameters from pretherapy ^18^F-FDG PET images in predicting response to therapy in breast, lung, cervix and head and neck cancers, reporting AUC values between 0.7 and 1.0 [22–25]. In particular, Cook et al. [25] carried out a Kaplan-Meier analysis to analyze the association between textural features and survival outcomes such as overall survival (OS), progression free survival (PFS) and local PFS. They reported sensitivity and specificity to have varied between 59% to 94% and 42% to 63%, respectively. The main limitation in these studies is the use of low sample sizes ranging from 9 to 53. Thus, the role of these metrics and the clinical relevance remains to be further validated.

From the results in Table 2, it is particularly interesting to notice the difference in performance between 1S-CNN and 3S-CNN. These results enhance the belief that local 3D information of the ^18^F-FDG uptake can be beneficial for the chemotherapy response prediction. Excluding LR, all the classifiers trained on texture features perform better when the feature vector is replaced by principal components–this is expected since several features contain redundant information. Also, the threshold SUVmax median outperformed all the algorithms except the 3S-CNN, revealing that the SUV carry predictive power. According to the rankings in Fig 5, the texture feature coarseness derived from NGTDMs is the parameter that best differentiates responders and non-responders. Coarseness describes local tumor texture based on differences between each voxel and the neighboring voxels in adjacent axial ^18^F-FDG PET images. This result is consistent with previously reported evidence that high coarseness values are associated with a greater risk of local tumor progression in non-small lung cancer [25]. Moreover, previous findings have also suggested that coarseness is a texture feature that may discriminate well between responders to chemoradiotherapy from non-responders in oesophageal cancer [21]. Remarkably, many texture features appearing in the top 10 ranking were extracted using a variety of different methods, including fractal analysis, statistical based texture matrices (including GLRLM, GLCM, GLZLSM, NGTDM) and the SUV parameter. This message stresses again the importance of including a very large ensemble of texture features in a radiomics approach. Finally, from the performance of SUVmax median threshold (see Table 2) and the importance ranking of the SUVmin (see Fig 5), our study supports the conflicting evidence that the SUV parameters can discriminate the behavior of a tumor to treatment before therapy.

The number of tumor volumes that is available for this study may lead to overfitting and consequently to degradation of an algorithm’s generalization ability on unseen test examples. The predictive algorithms we included in the comparison, such as GB, RF and SVM, encompass several mechanisms to prevent overfitting [52]. For both 3S-CNN and 1S-CNN several additional attempts were made to further reduce overfitting. For instance, we replaced the hyperbolic tangent non-linearities with rectified linear unit (ReLu) non-linearities. Compared to hyperbolic tangent non-linearities, ReLu accelerate the convergence of the MSGD algorithm for the training of the CNN and it is less prone to the gradient vanishing problem [56]. Also, we deployed the technique of Dropout in the FC layer of the CNN. Dropout prevents the neurons from co-adaptation, thus reducing the overfitting of the training dataset [57]. Despite the known advantages of these techniques, they did not significantly improve the generalization performance in our case.

Substantial improvements would be expected by increasing the number of training PET images for which we have clinical information. In future work will also aim at developing a multi-modality algorithm in order to take advantage from both PET and CT images, since PET images ignore the anatomical information and do not present well-defined tumor boundaries because of their relatively poor spatial resolution. We believe that a combination of anatomical and corresponding FDG uptake information will further improve the quality of extracted imaging features and lead to significant improvement in the neoadjuvant chemotherapy response prediction [58]. These model predictions could offer the potential to stratify patients for preoperative therapy before surgery in clinical trials.

## Conclusions

Esophageal cancer is associated with high mortality and it is of vital importance to be detected and treated in early stage. In advanced stages, preoperative chemotherapy or radiotherapy can play an essential role in the improvement of survival for patients who respond to the treatment. By contrast, for patients who do not respond to preoperative treatment there is a need for different treatment tactics in order to increase the probability of tumor control. Therefore, the ability to noninvasively predict treatment response before therapy is of great interest and could allow oncologists to personalize future cancer treatments in the clinic.

In the present study we have proposed two different methodologies to predict neoadjuvant chemotherapy response based on pretherapy ^18^F-FDG PET images. In the first methodology, 3S-CNN were employed to hierarchically learn FDG uptake patterns that are associated with response to neoadjuvant chemotherapy by Mandard. In the second methodology, a wide variety of “hand-engineered” features were derived from the same images and then used as predictor variables in machine learning algorithms. 3S-CNN algorithm outperformed all machine learning algorithms trained on “hand-engineered” ^18^F-FDG PET imaging features. In conjuction with the variety of the textural features ranked by GB algorithm, our preliminary results indicates that synthesizing features that extensively exploit the heterogeneity of the FDG uptake information with respect to chemotherapy response prediction might offer the potential to capture all the relevant information in the ^18^F-FDG PET images. However, further testing using larger datasets is required to validate the predictive power of 3S-CNN for clinical decision-making.
